# The Global Burden of Air Pollution on Mortality: The Need to Include Exposure to Household Biomass Fuel–Derived Particulates

**DOI:** 10.1289/ehp.1002397

**Published:** 2010-10

**Authors:** Jamie Rylance, Duncan G. Fullerton, Sean Semple, Jon G. Ayres

**Affiliations:** Liverpool School of Tropical Medicine, Liverpool, United Kingdom, E-mail: jrylance@liv.ac.uk; Scottish Centre for Indoor Air, Institute of Applied Health Sciences, University of Aberdeen, Aberdeen, United Kingdom; Institute of Occupational and Environmental Medicine, University of Birmingham, Edgbaston, Birmingham, United Kingdom

Anenberg et al. (2010) demonstrated that global mortality associated with outdoor ozone and particulate matter (PM) exposure has been underestimated and that anthropogenic atmospheric PM rather than ozone is the main contributor to death. Although we acknowledge that their investigation was concerned with outdoor air pollution alone, we feel that attention should be drawn to the burden of disease from household air pollution.

Half the world’s population is exposed to fine PM [< 2.5 μm in aerodynamic diameter (PM_2.5_)] in their own homes as a consequence of using biomass fuels such as wood, charcoal, and animal/crop residues for cooking, lighting, and heating. Such exposure is prolonged, extensive, and overlooked by examination of atmospheric models alone (Torres-Duque et al. 2008).

Combustion of biomass fuels has been repeatedly demonstrated to produce high concentrations of domestic air pollution, with PM_2.5_ exposures extending in to the milligram per cubic meter range, orders of magnitude above concentrations from exposure to anthropogenic particulate pollution outdoors (Regalado et al. 2006). Rural populations, and women in particular, are likely to have particularly high indoor exposures because of the extended time spent on cooking and household activity (Mestl et al. 2007).

Anenberg et al. (2010) used exposure– response functions derived from epidemiological studies of outdoor air, which emphasize cardiopulmonary mortality in older cohorts. Household air pollution from biomass fuel combustion contributes to chronic respiratory disease and cardiorespiratory events. However, it is particularly implicated in pneumonia in young children (Dherani et al. 2008) and has been ranked the 11th most important risk factor in global mortality, predominantly because of the association with infection (Ezzati et al. 2004). These early deaths would contribute considerably to the estimate of years of life lost due to PM.

We agree with Anenberg et al. (2010) that anthropogenic PM is an important global cause of premature death. However, outdoor levels report only part of the picture and may significantly underestimate the total PM-related mortality burden.

Recent work (Pope et al. 2009) has brought together data on exposure–response functions for outdoor air pollution and cigarette smoking, and there is a need for additional similar work to integrate studies on indoor biomass combustion (Ezzati et al. 2000). These studies would help clarify the exposure–response function of household air pollution as well as assist in the important process of identifying the most cost-efficient means of reducing exposure among the 3 billion people who bear the health burden from high particulate concentrations at home.
